# Quality of Life in Women with Gynecologic Cancer and Coexisting Chronic Conditions: The Role of Cancer-Related Fatigue and Perceived Caregiver Support

**DOI:** 10.3390/healthcare14193318

**Published:** 2026-10-05

**Authors:** Jie Bai, Guan Gui, Aina Chen, Tingting Chen, Xiaoyan Zhang, Ying Liu, Haiyan Zhou, Jianhui Gao, Fan Xu, Qiaosong Wang, Nuannuan Pan, Weina Jin, Hui Jiang

**Affiliations:** 1 Department of Gynecologic Oncology, Shanghai Key Laboratory of Maternal Fetal Medicine, Shanghai Institute of Maternal-Fetal Medicine and Gynecologic Oncology, Shanghai First Maternity and Infant Hospital, School of Medicine, Tongji University, Shanghai 200092, China; 2 Department of Gynecologic Oncology, The First Affiliated Hospital of Bengbu Medical University, Bengbu 233004, China; 3 Department of Obstetrics and Gynecology, Shanghai East Hospital, School of Medicine, Tongji University, Shanghai 200092, China; 4 Clinical Research Unit, Shanghai Key Laboratory of Maternal Fetal Medicine, Shanghai Institute of Maternal-Fetal Medicine and Gynecologic Oncology, Shanghai First Maternity and Infant Hospital, School of Medicine, Tongji University, Shanghai 200092, China; 5 Shanghai Key Laboratory of Maternal Fetal Medicine, Shanghai Institute of Maternal-Fetal Medicine and Gynecologic Oncology, Shanghai First Maternity and Infant Hospital, School of Medicine, Tongji University, Shanghai 200092, China

**Keywords:** gynecologic cancer, chronic illness, multimorbidity, cancer-related fatigue, caregiver support, quality of life

## Abstract

**Background:** Women with gynecologic cancer and coexisting chronic conditions may experience extensive symptom burden and impaired quality of life. However, the adjusted associations of cancer-related fatigue, multimorbidity, and perceived caregiver support with quality of life remain insufficiently understood. **Methods:** A cross-sectional study was conducted among 210 Chinese women with cervical, ovarian, endometrial, or vulvar cancer and at least one physician-diagnosed chronic condition. A structured questionnaire was used to collect socio-demographic data, clinical data, quality-of-life measures, cancer fatigue levels, and perceived caregiver support. Hierarchical linear regression models with robust standard errors were used to examine factors associated with quality of life. **Results:** The mean quality of life score was 62.16 (SD = 18.72), and the mean fatigue score was 26.80 (SD = 12.39). Ninety-two participants (43.8%) had two or more chronic conditions. Quality of life was strongly negatively correlated with fatigue (r = −0.78, *p* < 0.001) and positively correlated with perceived caregiver support (r = 0.33, *p* < 0.001). Demographic and clinical factors explained 26.2% of the variance in quality of life. Adding perceived caregiver support increased the explained variance to 28.6%, and greater support was associated with better quality of life (B = 3.45, *p* = 0.006). After fatigue was added, the explained variance increased to 68.0%. Greater fatigue was strongly associated with poorer quality of life (B = −1.11, *p* < 0.001), whereas perceived caregiver support was no longer significant (B = 0.40, *p* = 0.672). FIGO stage III–IV disease remained associated with lower quality of life (B = −4.39, *p* = 0.005). The association between multimorbidity and quality of life was attenuated in the final model (B= −3.11, *p* = 0.063). **Conclusions:** Cancer-related fatigue was strongly associated with poorer quality of life among women with gynecologic cancer and coexisting chronic conditions. Perceived caregiver support was associated with better quality of life before fatigue was included in the model, but this association was attenuated after adjustment for fatigue. These findings support routine fatigue assessment and integrated symptom management in supportive care for this population.

## 1. Introduction

Gynecologic cancers, including cervical, ovarian, and endometrial cancers, impose substantial physical, psychological, and social burdens on women. An estimated 1.4 million women were diagnosed with gynecological cancers globally in 2020, resulting in 680,000 deaths [1]. Although advances in cancer screening and treatment have improved survival, many patients experience persistent symptoms and functional limitations during and after treatment [2]. Growing evidence indicates the association between the heavy symptom burden and patients’ overall quality of life in the context of cancer [3]. Consequently, quality of life has become an important outcome in evaluating the overall well-being and supportive care needs of women with gynecologic cancer.

Elevated risks among cancer survivors include late therapy-related effects, accelerated senescence, and comorbid chronic diseases, ranging from cardiovascular conditions to diabetes and renal failure [4]. The coexistence of cancer and chronic conditions may increase treatment complexity, symptom burden, medication use, healthcare needs, and difficulties in daily functioning [5,6]. Chronic conditions may also restrict patients’ ability to tolerate cancer treatment and manage treatment-related symptoms. Women with coexisting chronic conditions may therefore be vulnerable to impaired quality of life.

Fatigue is one of the most common and distressing symptoms experienced by patients with cancer [7]. It may be impacted by patient characteristics encompassing sociodemographic background, clinical history, pre-existing comorbidities, lifestyle behaviors, and psychosocial status [8]. Persistent fatigue can interfere with self-care, social participation, treatment adherence, and daily activities, thereby negatively affecting multiple dimensions of quality of life and may be a risk factor for reduced survival. However, the extent to which fatigue is associated with quality of life among women simultaneously managing gynecologic cancer and chronic conditions remains insufficiently understood.

Perceived social support refers to an individual’s subjective appraisal of the availability and adequacy of supportive resources [9]. The conceptualization and operationalization of social support have varied considerably across studies. For example, some research has focused on dyadic interactions between care-recipients and their support networks [10]. From the care-recipient perspective, perceived support reflects not only the availability of assistance but also the extent to which individuals feel understood, encouraged, and able to rely on others when facing health-related challenges [11]. These pathways are thought to operate through behavioral, psychological, and emotional mechanisms that affect both the care-recipient and the caregiver.

Compared with broader social networks, primary caregivers often provide sustained instrumental and emotional assistance during cancer treatment and chronic disease management; therefore, caregiver-specific support may be particularly relevant for patients experiencing complex health needs. Emotional reassurance, practical assistance, and encouragement from caregivers may help patients manage symptoms, adhere to treatment, and adapt to changes in daily and family roles [12,13]. Therefore, greater perceived caregiver support may be associated with better quality of life among women managing both cancer and chronic health problems [14]. However, the relationship between caregiver support and quality of life may be intertwined with patients’ symptom burden, as fatigue and other cancer-related symptoms may influence both patients’ perceived needs for support and their overall well-being.

Most previous studies of quality of life among women with gynecologic cancer have examined cancer-related clinical factors or included patients irrespective of coexisting chronic conditions. Consequently, relatively little is known about how symptom burden and caregiver support are associated with quality of life among women simultaneously managing gynecologic cancer and chronic illness, particularly in China. The present study addresses this gap by focusing specifically on women with at least one chronic condition and by examining the incremental associations of perceived caregiver support and cancer-related fatigue with quality of life after accounting for demographic and clinical characteristics.

This study aimed to

(1) Describe the levels of fatigue, perceived caregiver support, and quality of life among Chinese women with gynecologic cancer and coexisting chronic conditions;
(2) Examine the associations of cancer-related fatigue and perceived caregiver support with quality of life;
(3) Identify demographic and clinical factors associated with quality of life after multivariable adjustment in this population.

## 2. Materials and Methods

### 2.1. Design

This was a multicenter, cross-sectional analysis of baseline data from an intervention study. Data were collected from January to December 2025 in the gynecology departments of three tertiary hospitals in Shanghai and Anhui Province, China. The reporting of this study followed the STROBE guidelines [15].

### 2.2. Sample

#### 2.2.1. Sample Size Calculation

The required sample size was estimated using G*Power version 3.1 for multiple linear regression. Assuming a medium effect size (f^2^ = 0.15), a two-sided significance level of 0.05, 90% power, and an anticipated model with 15 predictor parameters, at least 171 participants were required. Allowing for approximately 15% incomplete or invalid responses, the minimum recruitment target was set at 202 participants.

#### 2.2.2. Inclusion and Exclusion Criteria

Participants were eligible if they: (1) were aged 18 years or older; (2) had a histologically confirmed diagnosis of cervical, ovarian, endometrial, or vulvar cancer; (3) had at least one physician-diagnosed chronic condition, including hypertension, diabetes mellitus, cardiovascular disease, cerebrovascular disease, thyroid disease, or other long-term medical conditions; (4) provided informed consent.

Participants were excluded if they: (1) had severe cognitive, psychiatric, or communication difficulties that prevented questionnaire completion; (2) were critically ill or clinically unstable at the time of data collection; or (3) had incomplete data for the principal study variables.

### 2.3. Measures

#### 2.3.1. Quality of Life

Quality of life was assessed using the Functional Assessment of Cancer Therapy–General (FACT-G) [16]. The FACT-G is a 27-item cancer-specific quality-of-life instrument comprising four domains: physical well-being, social/family well-being, emotional well-being, and functional well-being. Each item is rated on a five-point Likert scale ranging from 0 (“not at all”) to 4 (“very much”). Negatively worded items were reverse-scored in accordance with the scoring manual, and domain scores were summed to generate a total score ranging from 0 to 108. Higher scores indicate better quality of life. The Chinese version of the FACT-G has demonstrated acceptable psychometric properties in patients with cancer [17]. In the present study, the FACT-G total score was used as the primary outcome. In this study, Cronbach’s alpha was 0.89.

#### 2.3.2. Fatigue

Cancer-related fatigue was assessed using the Cancer Fatigue Scale (CFS) [18]. The CFS contains 15 items across three dimensions: physical fatigue, affective fatigue, and cognitive fatigue. Each item is rated on a five-point Likert scale from 1 (“not at all”) to 5 (“very much”). Dimension scores are calculated according to the original scoring algorithm, including reverse scoring of specified items. The physical fatigue subscale ranges from 0 to 28, while the affective and cognitive fatigue subscales each range from 0 to 16. The total score ranges from 0 to 60, with higher scores indicating more severe cancer-related fatigue. The Chinese version of the CFS has demonstrated acceptable reliability and validity among patients with cancer. In the present study, the CFS total score was used in the analyses. In this study, Cronbach’s alpha was 0.81. 

#### 2.3.3. Perceived Caregiver Support

Perceived caregiver support was assessed using a single item asking participants to rate the degree of support they perceived from their primary caregiver. This item was developed for the parent intervention study to capture patients’ global perception of caregiver support. Responses were originally coded as 1 (“very high”), 2 (“high”), 3 (“moderate”), 4 (“low”), and 5 (“very low”). To facilitate interpretation, scores were reverse-coded so that higher values indicated greater perceived caregiver support, resulting in a score ranging from 1 to 5. This single item was intended to capture a global appraisal of caregiver support, and it had not undergone independent psychometric validation.

#### 2.3.4. Coexisting Chronic Conditions

We identified coexisting chronic conditions through self-report of physician-diagnosed chronic diseases. Participants reported both the number and type of chronic conditions. Reported conditions included hypertension, diabetes mellitus, dyslipidemia, coronary heart disease, cerebrovascular disease, thyroid disease, and other chronic illnesses. The number of chronic conditions was calculated for each participant. For the primary analysis, participants were categorized as having either one chronic condition or two or more chronic conditions. The latter group was defined as having multimorbidity. In a sensitivity analysis, the number of chronic conditions was entered as a continuous variable.

#### 2.3.5. Demographic and Clinical Characteristics

Demographic characteristics included age, body mass index, years of education, place of residence, marital status, employment status, monthly household income, and type of health insurance. Body mass index was calculated as weight in kilograms divided by height in meters squared. Marital status was dichotomized as married or not currently married. Employment status was recorded as full-time employment, flexible employment, unemployment, or retirement. For selected analyses, full-time and flexible employment were combined into an employed category.

Clinical characteristics included cancer type and International Federation of Gynecology and Obstetrics stage. Cancer type was classified as cervical, ovarian, endometrial, or vulvar cancer. Because only two participants had vulvar cancer, they were retained in the descriptive analysis but excluded from regression models involving cancer type. FIGO stage was recorded as stages I–IV. For the primary regression analysis, stage was categorized as early stage (I–II) or advanced stage (III–IV). A four-category FIGO-stage variable was examined in sensitivity analysis. All 210 participants were included in descriptive and primary bivariate analyses. Because only two participants had vulvar cancer, regression models that included cancer type were restricted to participants with cervical, ovarian, or endometrial cancer (*n* = 208) to avoid unstable estimates for the vulvar-cancer category.

### 2.4. Data Collection

Using a convenience sampling approach, the data were sourced from the baseline assessment of an intervention study. Potential participants were approached through long-term support, assistance, and healthcare follow-up programs at each hospital. We provided a brief introduction to the study’s purpose and aims, and willing participants were invited to complete a face-to-face survey during their routine rechecks or when they visited specifically for this study. In each setting, a trained research nurse was responsible for data collection. The primary author developed the questionnaire and standardized the introduction wording to maintain consistency across sites. Every two weeks, the primary author and the nurses responsible for data collection met to discuss any potential issues.

### 2.5. Data Analysis

The primary outcome was the FACT-G total score. Cancer-related fatigue and perceived caregiver support were treated as primary explanatory variables. No separate secondary outcome was defined for the present cross-sectional analysis.

Participant characteristics were summarized using frequencies and percentages for categorical variables and means with standard deviations or medians with interquartile ranges for continuous variables, depending on their distributions. Continuous variables were assessed for normality using histograms, Q–Q plots, and skewness and kurtosis statistics. 

Coexisting chronic conditions were described according to both the number and type of conditions. The number of chronic conditions was calculated for each participant and categorized as one condition or two or more conditions. For descriptive analyses, major chronic conditions were coded as separate dichotomous variables because participants could report more than one condition. Conditions with small frequencies were grouped into an “other chronic conditions” category for descriptive presentation. The primary multivariable analyses used multimorbidity status rather than individual chronic-condition indicators.

Pearson correlation coefficients were used to examine associations between approximately continuous variables, including FACT-G total score, fatigue, age, BMI, and years of education. Because perceived caregiver support was measured using a five-point ordinal item, its association with FACT-G was examined using Pearson correlation in the primary analysis and Spearman’s rank correlation in a sensitivity analysis.

Differences in quality-of-life scores across demographic and clinical subgroups were examined using independent-samples *t* tests or one-way analysis of variance, as appropriate. The Mann–Whitney U test or Kruskal–Wallis test was used when the assumptions of parametric tests were not met. Pearson’s or Spearman’s correlation coefficients were calculated to examine the associations among fatigue, perceived caregiver support, and quality of life.

Influential observations were evaluated using standardized residuals, leverage values, and Cook’s distance. Observations with Cook’s distance greater than 4/n were examined as potentially influential rather than automatically excluded. Model assumptions were further assessed using residual-versus-fitted plots and normal Q–Q plots.

Candidate covariates were considered based on clinical and theoretical relevance, subgroup size, and model stability. Age and education were retained as continuous demographic covariates, while marital status and household income were retained because of their clinical and psychosocial relevance. BMI, residence, and health insurance were not included in the final hierarchical models because they showed no meaningful bivariate association with quality of life. Employment status was not retained because of sparse categories and increased multicollinearity when included together with age and household income. Cancer type, FIGO stage, and multimorbidity status were retained as clinically relevant disease-related covariates. 

No imputation was performed. Participants with incomplete data for the principal study variables were excluded before analysis. All participants included in the analytic sample had complete data for the variables used in the descriptive and bivariate analyses. Regression analyses were conducted using complete cases; the reduction from 210 to 208 participants was due to exclusion of the two women with vulvar cancer from models containing cancer type rather than missing data.

Three hierarchical regression models were constructed. Model 1 included demographic and clinical covariates to establish a baseline adjusted model. Model 2 additionally included perceived caregiver support to examine whether this psychosocial factor was associated with quality of life beyond demographic and clinical characteristics. Model 3 subsequently added cancer-related fatigue to examine whether the associations observed in Model 2 remained after accounting for fatigue. The order of entry was specified to evaluate the incremental associations of caregiver support and fatigue and was not intended to imply a temporal or causal sequence.

Unstandardized regression coefficients (B), robust standard errors, 95% confidence intervals, standardized regression coefficients (β), and *p* values were reported. Changes in R^2^ were used to describe the additional variance explained by each block. Because robust standard errors were used, robust Wald F tests were used to evaluate the contribution of newly entered blocks as the robust-inference analogue of conventional F-change tests. Regression assumptions were evaluated by examining linearity, normality and homoscedasticity of residuals, multicollinearity, and influential observations. Variance inflation factor values below 5 were considered acceptable. Data analyses were performed using Stata 15.1. All statistical tests were two-sided, and a *p*-value of <0.05 was considered statistically significant. 

### 2.6. Ethical Considerations

The study was conducted in accordance with the Declaration of Helsinki and was approved by the Ethics Committee of Shanghai First Maternity and Infant Hospital (approval number: KS25471). Written informed consent was obtained from all participants before data collection. Participation was voluntary, confidentiality was maintained throughout the study, and participants were informed that their decision to participate or withdraw would not affect their clinical care.

## 3. Results

### 3.1. Participant Characteristics and Levels of Key Variables

A total of 224 potentially eligible participants were approached. Fourteen were excluded because of incomplete data or non-completion of the questionnaire, leaving 210 participants in the final analytic sample. Their mean age was 63.71 years (SD = 7.98). The sample included 66 participants with cervical cancer (31.4%), 74 with ovarian cancer (35.2%), 68 with endometrial cancer (32.4%), and 2 with vulvar cancer (1.0%). Overall, 118 participants (56.2%) had one chronic condition, whereas 92 (43.8%) had two or more chronic conditions. Hypertension was the most common coexisting chronic condition (*n* = 154, 73.3%), followed by diabetes mellitus (*n* = 54, 25.7%), dyslipidemia (*n* = 34, 16.2%), coronary heart disease (*n* = 30, 14.3%), cerebrovascular disease (*n* = 18, 8.6%), and thyroid disease (*n* = 10, 4.8%).

Most participants reported very high or high caregiver support (*n* = 150, 71.4%), while 54 (25.7%) reported moderate support and 6 (2.9%) reported low or very low support. The mean fatigue score was 26.80 (SD = 12.39), and the mean FACT-G total score was 62.16 (SD = 18.72). Table 1 summarizes the demographic and clinical characteristics of the study participants.

### 3.2. Bivariate Associations with Quality of Life

The FACT-G total score was strongly and negatively correlated with fatigue (r = −0.78, *p* < 0.001) and positively correlated with perceived caregiver support (r = 0.33, *p* < 0.001). Older age was weakly associated with poorer quality of life (r = −0.20, *p* = 0.004). Body mass index and years of education were not significantly associated with the FACT-G total score.

Participants with two or more chronic conditions reported significantly poorer quality of life than those with one chronic condition (56.20 ± 18.17 vs. 66.81 ± 17.88; *p* < 0.001). Participants with FIGO stage III–IV disease also had lower FACT-G scores than those with stage I–II disease (59.06 ± 18.67 vs. 64.88 ± 18.43; *p* = 0.024). Quality of life did not differ significantly according to marital status or place of residence.

Among participants with cervical, ovarian, or endometrial cancer, quality of life differed significantly by cancer type (*p* = 0.008). Those with endometrial cancer had lower FACT-G scores than those with cervical cancer (B = −8.15, 95% CI [−13.93, −2.37], *p* = 0.006), whereas no significant difference was observed between ovarian and cervical cancer. Household income and employment status were also associated with quality of life in unadjusted analyses. Bivariate associations with quality of life are shown in Table 2.

### 3.3. Multivariable Associations with Quality of Life

The hierarchical regression analyses included 208 participants because the two participants with vulvar cancer were excluded from models containing cancer type. In Model 1, demographic and clinical characteristics explained 26.2% of the variance in FACT-G total scores. More years of education were associated with higher FACT-G scores, whereas having two or more chronic conditions, FIGO stage III–IV disease, selected household income categories, and endometrial cancer were associated with lower FACT-G scores.

The addition of perceived caregiver support in Model 2 increased the explained variance to 28.6% (ΔR^2^ = 0.024; robust Wald F = 7.76, *p* = 0.006). Greater perceived caregiver support was associated with higher FACT-G scores (B = 3.45, 95% CI [1.01, 5.90], β = 0.175, *p* = 0.006).

After cancer-related fatigue was added in Model 3, the explained variance increased to 68.0% (ΔR^2^ = 0.395; robust Wald F = 214.81, *p* < 0.001). Greater fatigue was strongly associated with lower FACT-G scores (B = −1.11, 95% CI [−1.26, −0.96], β = −0.734, *p* < 0.001). The association between perceived caregiver support and FACT-G score was attenuated and was no longer statistically significant (B = 0.40, 95% CI [−1.47, 2.27], β = 0.020, *p* = 0.672). FIGO stage III–IV remained associated with lower FACT-G scores (B = −4.39, *p* = 0.005), whereas married status was associated with higher scores (B = 6.09, *p* = 0.041). Compared with cervical cancer, ovarian cancer was associated with higher adjusted FACT-G scores (B = 7.84, *p* < 0.001). The coefficient for multimorbidity was attenuated and did not reach statistical significance in the final model (B = −3.11, *p* = 0.063). Full regression results are presented in Table 3.

Visual inspection of the residual-versus-fitted and normal Q–Q plots indicated no major violations of linearity or residual normality.

### 3.4. Sensitivity Analyses

Sensitivity analyses were broadly consistent with the primary finding regarding cancer-related fatigue. When the number of chronic conditions was entered as a continuous variable, it was not significantly associated with quality of life (B = −1.19, 95% CI [−3.14, 0.76], *p* = 0.229). Fatigue remained strongly associated with lower FACT-G scores (B = −1.12, *p* < 0.001). The directions of the associations for advanced-stage disease, marital status, the highest household income category, and ovarian cancer were consistent with the primary model, although the magnitude and statistical significance of individual coefficients varied across models and sensitivity analyses.

Because perceived caregiver support was measured using a five-point ordinal item, a sensitivity analysis using Spearman’s rank correlation was conducted. The association remained statistically significant (ρ = 0.323, *p* < 0.001), consistent with the Pearson correlation observed in the primary analysis (r = 0.334, *p* < 0.001).

In an expanded sensitivity model additionally adjusting for BMI, residence, employment status, and health insurance, fatigue remained strongly associated with lower FACT-G scores (B = −1.08, *p* < 0.001), whereas perceived caregiver support remained statistically non-significant (B = 1.58, *p* = 0.112). Several categorical predictors in the expanded model had sparse subgroup sizes and higher variance inflation factors, particularly variables related to employment, residence, and insurance. These findings were therefore treated as sensitivity analyses, while the more parsimonious hierarchical model was retained as the primary analysis.

When FIGO stage was modeled using four categories, the overall association between stage and quality of life remained statistically significant (*p* = 0.004). Compared with stage I disease, stage III disease was associated with a lower FACT-G score (B = −7.51, 95% CI [−11.70, −3.32], *p* = 0.001), whereas the coefficients for stages II and IV were not statistically significant. Fatigue remained strongly associated with poorer quality of life (B = −1.12, *p* < 0.001). See Supplementary Table S1.

To further examine the potential conceptual overlap between cancer-related fatigue and the FACT-G, additional domain-specific sensitivity analyses were conducted using the four FACT-G domains as separate outcomes. After adjustment for age, education, multimorbidity status, FIGO stage, marital status, household income, cancer type, and perceived caregiver support, fatigue remained significantly associated with all four domains. The standardized associations were strongest for physical well-being (β = −0.677, *p* < 0.001) and functional well-being (β = −0.660, *p* < 0.001), while substantial associations were also observed for social/family well-being (β = −0.548, *p* < 0.001) and emotional well-being (β = −0.505, *p* < 0.001). See Supplementary Table S2.

## 4. Discussion

This study investigated quality of life and its correlates among women with gynecologic cancer and at least one coexisting chronic condition. Three key findings were identified. First, cancer-related fatigue showed a strong and consistent association with poorer quality of life and accounted for a substantial increase in the variance explained by the regression model. Second, perceived caregiver support was associated with better quality of life before fatigue was entered into the model, but this association was markedly attenuated after adjustment for fatigue. Third, advanced-stage disease remained associated with poorer quality of life after adjustment, whereas the association between multimorbidity and quality of life became weaker after fatigue was considered. Together, these findings indicate that fatigue was more strongly associated with quality of life than the number of coexisting chronic conditions in this cross-sectional sample.

In this study, the participants experienced a considerable burden of both cancer and chronic disease. Nearly half had two or more chronic conditions, and hypertension, diabetes, dyslipidemia, coronary heart disease, and cerebrovascular disease were common. This pattern is consistent with the older age profile of the sample and highlights the clinical complexity of women receiving care for gynecologic cancer while simultaneously managing non-cancer conditions. In comparison with a prior study conducted in Shanxi [19], which reported a mean FACT-G total score of 70.74 ± 2.43 among gynecological cancer patients at one week post-surgery, the quality-of-life scores in our sample were notably lower. Our observed mean FACT-G score was 62.16. The difference from the Shanxi study may reflect variation in sample characteristics, including age, cancer type, treatment status, disease stage, and the presence of chronic conditions. However, direct comparisons with other studies should be made cautiously because FACT-G scores vary according to cancer type, treatment status, disease stage, age, and the prevalence of chronic conditions. Unlike studies that include gynecologic cancer populations irrespective of comorbidity, the present analysis focused specifically on women with at least one chronic condition, a group that may face overlapping treatment demands, symptom burden, and functional limitations.

Cancer-related fatigue has consistently been associated with poorer quality of life in cancer populations [20]. The strong inverse correlation between CFS and FACT-G scores and the substantial increase in explained variance after fatigue was entered highlight the close relationship between fatigue and quality of life. However, the magnitude of this association should be interpreted cautiously. Because the FACT-G includes physical and functional well-being domains that are conceptually related to fatigue, part of the observed association and increase in explained variance may reflect conceptual overlap between the two instruments. Each one-point increase in fatigue was associated with an approximately 1.1-point reduction in the FACT-G total score after adjustment for demographic and clinical factors. Although the cross-sectional design does not establish directionality, the magnitude and consistency of this association indicate that fatigue assessment should be central to supportive care for women with gynecologic cancer and chronic conditions. Cancer-related fatigue is a multifaceted clinical phenomenon, with its perceived impact and limitations varying considerably among individuals [21]. Unlike the transient fatigue experienced by healthy individuals in response to daily activities, cancer-related fatigue is characterized by persistent physical exhaustion, weakness, and pronounced tiredness that occur with minimal exertion or after only brief periods of activity. The etiology of fatigue is complex, potentially arising from the interplay of cancer itself, treatment-related side effects, comorbid chronic conditions, physical deconditioning, sleep disturbances, nutritional deficits, and psychological distress [22]. As such, its optimal management likely necessitates comprehensive, multidimensional assessment rather than a single-modality approach. 

Perceived caregiver support was positively associated with quality of life in the bivariate analysis and remained significant after adjustment for demographic and clinical characteristics. However, this association attenuated after including cancer-related fatigue in the final model. This finding should be interpreted cautiously. Caregiver support was assessed using a single global item that had not undergone independent psychometric validation and may not adequately capture emotional, instrumental, informational, and appraisal dimensions of support. Moreover, 71.4% of participants selected one of the two highest support categories, and 46.7% selected the maximum score, indicating restricted variability and substantial concentration at the upper end of the scale. The attenuation of the association in the final model may therefore reflect, at least in part, limited measurement sensitivity and restricted range rather than evidence that caregiver support is clinically unimportant. In addition, because the data were collected at one time point, the relationships among fatigue, perceived support, and quality of life cannot be temporally ordered, and the findings should not be interpreted as demonstrating mediation. Future studies should use validated multidimensional caregiver- or social-support measures and longitudinal designs to clarify these relationships.

Women with two or more chronic conditions had substantially lower unadjusted quality-of-life scores than those with one condition. This pattern is consistent with previous evidence showing poorer quality of life with increasing multimorbidity burden [23]. In the present study, the association remained significant before fatigue was included but was attenuated in the final model. Similarly, the continuous number of chronic conditions was not significantly associated with quality of life after multivariable adjustment in the sensitivity analysis. These findings suggest that simple condition counts may incompletely capture the clinical burden associated with multimorbidity, which may also reflect symptom severity, functional consequences, treatment complexity, and disease-specific burden. Future studies should therefore consider weighted multimorbidity indices, disease severity, medication burden, treatment complexity, and condition-specific symptoms.

In accordance with earlier research involving adults with breast, lung, pancreatic, and colorectal cancers [24], advanced-stage disease remained associated with lower quality of life after multivariable adjustment. Specifically, women with FIGO stage III–IV disease had lower adjusted FACT-G scores than those with stage I–II disease. When FIGO stage was modeled using four categories, the overall association with FACT-G score remained statistically significant. The largest coefficient was observed for stage III disease, whereas the stage IV estimate was not statistically significant. The latter should not be interpreted as evidence of similar quality of life between stage IV and stage I, given the smaller subgroup size and potential sampling variability. More advanced disease may be associated with greater symptom burden, treatment intensity, uncertainty, and functional limitations, although these mechanisms were not directly examined in the present study. 

Several other adjusted associations require cautious interpretation. Married women had higher quality-of-life scores than unmarried women in the final model, possibly reflecting the practical, emotional, or financial resources associated with marriage [25]. This is broadly consistent with findings from the United States suggesting that married adults may report greater family support [26]. However, the non-married group was small and heterogeneous, including divorced, never-married, and widowed participants. The adjusted advantage observed for ovarian cancer compared with cervical cancer also emerged only after fatigue and other covariates were included. This change from the unadjusted analysis suggests substantial confounding by symptom burden, stage, or other clinical characteristics rather than an inherently better quality of life among women with ovarian cancer. Similarly, the lower adjusted quality of life observed in the highest-income group was contrary to conventional expectations. Because this subgroup included only eight participants, the estimate was potentially unstable and should be treated as exploratory rather than as evidence that higher income is detrimental.

The magnitude of the association between cancer-related fatigue and overall FACT-G scores should be interpreted in light of some conceptual overlap between the instruments, particularly between fatigue and the physical and functional well-being domains of the FACT-G. In additional domain-specific sensitivity analyses, fatigue showed the strongest adjusted associations with physical and functional well-being. However, significant associations were also observed with social/family and emotional well-being. These findings suggest that conceptual overlap may partly contribute to the strong association between fatigue and overall quality of life, but does not fully account for the observed relationship.

The findings have several clinical implications. These findings support routine assessment of cancer-related fatigue as part of supportive care for women managing gynecologic cancer and coexisting chronic conditions. Because fatigue may reflect interacting oncological, treatment-related, and non-oncological factors, its clinical evaluation should consider potentially contributing symptoms and comorbid conditions within a multidisciplinary framework.

### Strengths and Limitations

This study has several strengths. It focused on an understudied subgroup of women managing both gynecologic cancer and chronic illness, included multiple cancer types and disease stages, and examined fatigue, caregiver support, and clinical factors simultaneously. The use of hierarchical regression clarified how the association of caregiver support with quality of life changed after fatigue was considered. Robust standard errors and sensitivity analyses using alternative definitions of multimorbidity and cancer stage also supported the stability of the main fatigue finding.

Several limitations should be noted. First, the cross-sectional design precluded assessment of temporal ordering and changes in fatigue, perceived caregiver support, and quality of life over time; therefore, causal or mediational interpretations cannot be made. Second, chronic conditions were based on reported physician diagnoses, and the analysis did not account for disease severity, duration, control, complications, medication burden, or treatment complexity. Third, perceived caregiver support was assessed using a single non-validated global item, which did not capture the multidimensional nature or source of support and showed restricted variability with substantial concentration at the upper end of the scale. Fourth, the clinical sample included very few women with vulvar cancer, requiring their exclusion from regression models involving cancer type and limiting generalizability to this subgroup. Fifth, several categorical subgroups, particularly the highest-income and employed groups, were small, and their estimates should therefore be interpreted cautiously. Sixth, potentially relevant factors, including treatment phase, time since diagnosis, recurrence status, physical functioning, pain, sleep disturbance, nutritional status, and psychological distress, were not available for the present analysis and may represent sources of residual confounding. Finally, conceptual overlap between cancer-related fatigue and the physical and functional domains of the FACT-G may have contributed to the strong association between fatigue and total quality-of-life scores. However, domain-specific sensitivity analyses showed that fatigue was also associated with social/family and emotional well-being, suggesting that the observed relationship was not confined to the domains with the greatest conceptual overlap.

## 5. Conclusions

In conclusion, cancer-related fatigue was strongly associated with poorer quality of life among women with gynecologic cancer and coexisting chronic conditions. Perceived caregiver support was associated with better quality of life before fatigue was included in the model, but this association was attenuated after adjustment for fatigue. Advanced-stage disease also remained associated with poorer quality of life, whereas the association with multimorbidity was weaker in the fully adjusted model. These findings support routine fatigue assessment as part of integrated supportive care for this population. Clinical assessment should consider potentially contributing factors such as treatment effects, chronic disease control, nutritional status, psychosocial needs and others. Future longitudinal studies using validated multidimensional measures of caregiver support are needed to clarify the temporal relationships among fatigue, support, multimorbidity, and quality of life.

## Figures and Tables

**Table 1:** Demographic and clinical characteristics of participants (*n* = 210).

Characteristic	*n* (%) or Mean ± SD/Median (IQR)
**Age, years**	63.71 ± 7.98
**Body mass index, kg/m^2^**	25.43 ± 3.63
**Education, years**	7.16 ± 4.69
**Cancer type**	
Cervical cancer	66 (31.4)
Ovarian cancer	74 (35.2)
Endometrial cancer	68 (32.4)
Vulvar cancer	2 (1.0)
**FIGO stage**	
Stage I	56 (26.7)
Stage II	56 (26.7)
Stage III	66 (31.4)
Stage IV	32 (15.2)
**Residence**	
Shanghai	86 (41.0)
Other city	64 (30.5)
Rural area	60 (28.6)
**Marital status**	
Married	186 (88.6)
Not currently married	24 (11.4)
**Monthly household income, RMB**	
<5000	98 (46.7)
5000–9999	52 (24.8)
10,000–19,999	52 (24.8)
≥20,000	8 (3.8)
**Employment status**	
Full-time employed	6 (2.9)
Flexible employment	6 (2.9)
Unemployed	72 (34.3)
Retired	126 (60.0)
**Health insurance**	
Employee medical insurance	76 (36.2)
Resident medical insurance	44 (21.0)
New Rural Cooperative Medical Scheme	86 (41.0)
Commercial insurance	4 (1.9)
**Number of chronic conditions**	1 (1–2)
One chronic condition	118 (56.2)
Two or more chronic conditions	92 (43.8)
**Major coexisting chronic conditions ^a^**	
Hypertension	154 (73.3)
Diabetes mellitus	54 (25.7)
Dyslipidemia	34 (16.2)
Coronary heart disease	30 (14.3)
Cerebrovascular disease	18 (8.6)
Thyroid disease	10 (4.8)
**Perceived caregiver support**	4 (3–5)
Very high	98 (46.7)
High	52 (24.8)
Moderate	54 (25.7)
Low	4 (1.9)
Very low	2 (1.0)
**Fatigue score**	26.80 ± 12.39
**FACT-G total score**	62.16 ± 18.72

Note. Data are presented as *n* (%), mean ± standard deviation, or median (interquartile range), as appropriate. FIGO = International Federation of Gynecology and Obstetrics; FACT-G = Functional Assessment of Cancer Therapy–General. Perceived caregiver support was reverse-coded for analysis so that higher scores indicated greater perceived support. ^a^ Participants could report more than one chronic condition; therefore, percentages do not sum to 100%.

**Table 2:** Bivariate associations of demographic and clinical characteristics, fatigue, and perceived caregiver support with quality of life (*n* = 210).

Variable	FACT-G Total Score, Mean ± SD/Correlation Coefficient	*p* Value
**Continuous variables**		
Fatigue score	(r = −0.781)	<0.001
Perceived caregiver support	(r = 0.334)	<0.001
Age, years	(r = −0.197)	0.004
Body mass index, kg/m^2^	(r = 0.081)	0.243
Education, years	(r = 0.043)	0.540
**Number of chronic conditions**		<0.001
One chronic condition	66.81 ± 17.88	
Two or more chronic conditions	56.20 ± 18.17	
**FIGO stage**		0.024
Stage I–II	64.88 ± 18.43	
Stage III–IV	59.06 ± 18.67	
**Marital status**		0.196
Not currently married	57.50 ± 19.33	
Married	62.76 ± 18.61	
**Cancer type ^a^**		0.008
Cervical cancer	64.09 ± 14.55	
Ovarian cancer	65.11 ± 19.75	
Endometrial cancer	55.94 ± 19.12	
**Residence**		0.388
Shanghai	60.28 ± 19.88	
Other city	62.91 ± 21.28	
Rural area	64.07 ± 13.39	
**Monthly household income, RMB**		<0.001
<5000	65.37 ± 15.49	
5000–9999	64.81 ± 20.61	
10,000–19,999	54.96 ± 21.45	
≥20,000	52.50 ± 3.07	
**Employment status ^b^**		0.006
Employed	61.33 ± 21.55	
Unemployed	67.39 ± 15.25	
Retired	59.25 ± 19.68	
**Health insurance**		0.068
Employee medical insurance	57.66 ± 19.49	
Resident medical insurance	63.82 ± 22.33	
New Rural Cooperative Medical Scheme	65.26 ± 13.82	
Commercial insurance	63.00 ± 39.26	

Note. FACT-G = Functional Assessment of Cancer Therapy–General; FIGO = International Federation of Gynecology and Obstetrics. Pearson correlation coefficients are presented for approximately continuous variables in the primary bivariate analysis. A Spearman rank-correlation sensitivity analysis was additionally conducted for perceived caregiver support. Independent-samples *t* tests were used for binary variables. Overall *p* values for cancer type, residence, household income, employment status, and health insurance were obtained using regression models with robust standard errors. ^a^ The overall cancer-type comparison excluded two participants with vulvar cancer because of the very small subgroup size. ^b^ Full-time and flexibly employed participants were combined into the employed category.

**Table 3:** Hierarchical linear regression analyses of factors associated with quality of life among women with gynecologic cancer and coexisting chronic conditions (*n* = 208).

Variable	Model 1		Model 2		Model 3	
	B (Robust SE; 95% CI)	Β	B (Robust SE; 95% CI)	β	B (Robust SE; 95% CI)	β
Age, years	−0.20 (0.17; −0.53 to 0.13)	−0.087	−0.20 (0.16; −0.52 to 0.12)	−0.086	0.03 (0.13; −0.23 to 0.28)	0.011
Education, years	0.90 (0.32; 0.28 to 1.53) **	0.231	0.81 (0.30; 0.21 to 1.41) **	0.207	0.17 (0.21; −0.24 to 0.58)	0.043
Multimorbidity status	—	—	—	—	—	—
One chronic condition	Reference	—	Reference	—	Reference	—
Two or more chronic conditions	−6.06 (2.54; −11.07 to −1.04) *	−0.164	−4.86 (2.41; −9.62 to −0.11) *	−0.131	−3.11 (1.67; −6.40 to 0.17)	−0.084
FIGO stage	—	—	—	—	—	—
Stage I–II	Reference	—	Reference	—	Reference	—
Stage III–IV	−4.99 (2.14; −9.20 to −0.78) *	−0.136	−5.21 (2.09; −9.33 to −1.09) *	−0.141	−4.39 (1.54; −7.43 to −1.35) **	−0.119
Marital status	—	—	—	—	—	—
Not currently married	Reference	—	Reference	—	Reference	—
Married	4.20 (3.95; −3.58 to 11.99)	0.073	2.13 (3.93; −5.63 to 9.88)	0.037	6.09 (2.96; 0.25 to 11.94) *	0.106
Monthly household income, RMB	—	—	—	—	—	—
<5000	Reference	—	Reference	—	Reference	—
5000–9999	−5.76 (2.88; −11.44 to −0.08) *	−0.136	−6.37 (2.87; −12.03 to −0.70) *	−0.150	−3.20 (2.01; −7.16 to 0.76)	−0.075
10,000–19,999	−17.70 (3.40; −24.39 to −11.00) ***	−0.412	−15.89 (3.49; −22.77 to −9.01) ***	−0.370	−5.02 (2.76; −10.47 to 0.43)	−0.117
≥20,000	−19.14 (5.22; −29.44 to −8.84) ***	−0.200	−14.87 (6.34; −27.38 to −2.37) *	−0.156	−13.18 (3.71; −20.50 to −5.87) ***	−0.138
Cancer type	—	—	—	—	—	—
Cervical cancer	Reference	—	Reference	—	Reference	—
Ovarian cancer	3.07 (2.50; −1.86 to 8.01)	.080	2.53 (2.42; −2.24 to 7.31)	0.066	7.84 (1.99; 3.91 to 11.77) ***	0.204
Endometrial cancer	−6.70 (2.69; −12.01 to −1.40) *	−0.171	−6.46 (2.70; −11.78 to −1.13) *	−0.165	0.85 (1.82; −2.73 to 4.43)	0.022
Perceived caregiver support	—	—	3.45 (1.24; 1.01 to 5.90) **	0.175	0.40 (0.95; −1.47 to 2.27)	0.020
Cancer-related fatigue	—	—	—	—	−1.11 (0.08; −1.26 to −0.96) ***	−0.734
Constant	76.93 (13.29; 50.72 to 103.15) ***	—	64.18 (14.35; 35.87 to 92.49) ***	—	84.88 (9.83; 65.48 to 104.27) ***	—
N	208		208		208	
R^2^	0.262		0.286		0.680	
Adjusted R^2^	0.224		0.246		0.661	
ΔR^2^	—		0.024		0.395	
Overall robust F	9.13		8.97		35.37	
*p* value	<0.001		<0.001		<0.001	
Robust Wald F for added block	—		7.76		214.81	
*p*-value for added block	—		0.006		<0.001	

Note. B = unstandardized regression coefficient; SE = standard error; CI = confidence interval; β = standardized regression coefficient; FIGO = International Federation of Gynecology and Obstetrics. Model 1 included demographic and clinical covariates. Model 2 additionally included perceived caregiver support. Model 3 additionally included cancer-related fatigue. Robust standard errors were used. The robust Wald F test for the added block evaluates the contribution of the variable newly entered at each hierarchical step. Standardized coefficients for categorical predictors are reported for completeness but should be interpreted with caution because they depend on indicator coding and group prevalence. Two participants with vulvar cancer were excluded because of the very small subgroup size. * *p* < 0.05, ** *p* < 0.01, *** *p* < 0.001.

## Data Availability

The raw data supporting the conclusions of this article will be made available by the authors on request.

## Supplementary Materials

The following supporting information can be downloaded at: https://www.mdpi.com/article/10.3390/healthcare14193318/s1; Table S1: Sensitivity analyses of factors associated with quality of life (*n* = 208); Table S2: Domain-specific sensitivity analyses of the association between cancer-related fatigue and FACT-G domains (*n* = 208).
