# Supplementary material for: Quality of Life in Women with Gynecologic Cancer and Coexisting Chronic Conditions: The Role of Cancer-Related Fatigue and Perceived Caregiver Support
Source: Healthcare (Basel). 2026 Oct 5;14(19):3318. doi: 10.3390/healthcare14193318 (PMC13665012; doi:10.3390/healthcare14193318)
Supplement: Supplementary file 1 [file healthcare-14-03318-s001.zip › healthcare-4514013-supplementary.pdf]

Supplementary Table S1. Sensitivity analyses of factors associated with quality of life (N = 208)

Variable	Chronic-condition count model, B (SE)	Four-category FIGO-stage model, B (SE)
Age, years	0.03 (0.13)	0.05 (0.13)
Education, years	0.19 (0.21)	0.07 (0.21)
Number of chronic conditions	−1.19 (0.99)	—
**Multimorbidity status**		
One chronic condition	—	Reference
Two or more chronic conditions	—	−3.59 (1.66)*
**FIGO stage**		
Stage I–II	Reference	—
Stage III–IV	−4.70 (1.52)**	—
Stage I	—	Reference
Stage II	—	−2.98 (2.31)
Stage III	—	−7.51 (2.12)***
Stage IV	—	−2.55 (2.49)
**Marital status**		
Not currently married	Reference	Reference
Married	6.12 (2.99)*	5.60 (3.00)
**Monthly household income, RMB**		
<5,000	Reference	Reference
5,000–9,999	−3.27 (2.03)	−2.90 (2.00)
10,000–19,999	−5.04 (2.76)	−5.38 (2.70)*
≥20,000	−13.14 (3.68)***	−11.83 (4.19)**
**Cancer type**		
Cervical cancer	Reference	Reference
Ovarian cancer	7.67 (1.99)***	7.37 (2.01)***
Endometrial cancer	0.81 (1.84)	0.04 (1.85)
Perceived caregiver support	0.37 (0.98)	0.90 (1.01)
Fatigue	−1.12 (0.07)***	−1.12 (0.08)***
Constant	85.82 (10.45)	84.55 (9.94)
(R^2)	0.678	0.689
Overall FIGO-stage test	—	($F(3,193)=4.67$,\ p=.004)

Note. Two participants with vulvar cancer were excluded from these regression models because of the very small subgroup size.

Supplementary Table S2. Domain-specific sensitivity analyses of the association between cancer-related fatigue and FACT-G domains (N = 208)

FACT-G domain	Fatigue B (95% CI)	Standardized β	*p* value	Model R^2
Physical well-being	−0.371 (−0.423 to −0.320)	−0.677	<0.001	0.627
Social/family well-being	−0.229 (−0.278 to −0.179)	−0.548	<0.001	0.390
Emotional well-being	−0.170 (−0.220 to −0.120)	−0.505	<0.001	0.382
Functional well-being	−0.341 (−0.403 to −0.278)	−0.660	<0.001	0.615

Note. Each model adjusted for age, education, multimorbidity status, FIGO stage, marital status, household income, cancer type, and perceived caregiver support. Robust standard errors were used. Two participants with vulvar cancer were excluded from these regression models because of the very small subgroup size.
